# Impact of a high-density grid catheter on long-term outcomes for structural heart disease ventricular tachycardia ablation

**DOI:** 10.1007/s10840-020-00918-4

**Published:** 2021-01-04

**Authors:** Riccardo Proietti, Rory Dowd, Lim Ven Gee, Shamil Yusuf, Sandeep Panikker, Sajad Hayat, Faizel Osman, Kiran Patel, Handi Salim, Bashar Aldhoon, Will Foster, Ahmed Merghani, Michael Kuehl, Prithwish Banerjee, Nicolas Lellouche, Tarvinder Dhanjal

**Affiliations:** 1 Department of Cardiology, University Hospital Coventry & Warwickshire NHS Trust, Clifford Bridge Road, Coventry, CV2 2DX UK; 2 Department of Cardiac, Thoracic, Vascular Sciences, and University of Padua, Padua, Italy; 3 University of Warwick (Medical School), Gibbet Hill, Coventry, CV4 7AJ UK; 4 Hopital Henri Mondor Albert Chenevier, University Paris Est Creteil Paris XII, Avenue du Marechal de Lattre de Tassigny, 94000, Creteil, Inserm U955, Paris, France

**Keywords:** Ventricular tachycardia, Catheter ablation, High density mapping, Long term outcome

## Abstract

**Background:**

Substrate mapping has highlighted the importance of targeting diastolic conduction channels and late potentials during ventricular tachycardia (VT) ablation. State-of-the-art multipolar mapping catheters have enhanced mapping capabilities. The purpose of this study was to investigate whether long-term outcomes were improved with the use of a HD Grid mapping catheter combining complementary mapping strategies in patients with structural heart disease VT.

**Methods:**

Consecutive patients underwent VT ablation assigned to either HD Grid, Pentaray, Duodeca, or point-by-point (PbyP) RF mapping catheters. Clinical endpoints included recurrent anti-tachycardia pacing (ATP), appropriate shock, asymptomatic non-sustained VT, or all-cause death.

**Results:**

Seventy-three procedures were performed (33 HD Grid, 22 Pentaray, 12 Duodeca, and 6 PbyP) with no significant difference in baseline characteristics. Substrate mapping was performed in 97% of cases. Activation maps were generated in 82% of HD Grid cases (Pentaray 64%; Duodeca 92%; PbyP 33% (*p* = 0.025)) with similar trends in entrainment and pace mapping. Elimination of all VTs occurred in 79% of HD Grid cases (Pentaray 55%; Duodeca 83%; PbyP 33% (*p* = 0.04)). With a mean follow-up of 372 ± 234 days, freedom from recurrent ATP and shock was 97% and 100% respectively in the HD Grid group (Pentaray 64%, 82%; Duodeca 58%, 83%; PbyP 33%, 33% (log rank *p* = 0.0042, *p* = 0.0002)).

**Conclusions:**

This study highlights a step-wise improvement in survival free from ICD therapies as the density of mapping capability increases. By using a high-density mapping catheter and combining complementary mapping strategies in a strict procedural workflow, long-term clinical outcomes are improved.

## Introduction

Ventricular tachycardia (VT) ablation strategies for scar-dependent monomorphic VT have evolved from traditional entrainment and pace mapping to include activation and substrate mapping with or without local abnormal ventricular activity (LAVA) potential and decrementing evoked potential (DEEP) mapping in either paced or sinus rhythm [1, 2]. Substrate-guided approaches involve (1) the identification of dense scar, borderzone (BZ), and areas of functional block; (2) diastolic conduction channels (CC) identified with ripple mapping, voltage scanning, and/or DEEP potential mapping; and (3) LAVA potential mapping [1, 3–6]. These approaches individually have been shown to improve long-term outcome [7].

Prerequisites of substrate-guided approaches include detailed scar definition and mapping for near field low bipolar voltage fractionated signals with relatively slow conduction velocities, that help to define the VT site of origin and CCs [8–10]. In practice, multiple substrate and VT isthmus defining approaches are used in any given case to define the VT substrate, and long-term outcome data systematically combining these complementary approaches is lacking.

More recently, state-of-the-art multi-electrode mapping catheters have not only substantially decreased the time for point acquisition but have also facilitated high-density mapping. In addition to increased resolution of the VT substrate, multipolar mapping catheters have overcome the limitations of point-by-point (PbyP) RF catheter mapping, with an increase of near-field signals in the areas of interest and a decrease in far-field signals [11]. Increasing point density better defines the VT substrate and multipolar mapping catheters not only enhance map density but also provide wavefront directionality assessment [12, 13]. We have previously shown in the Omnimapping study that the unique electrode and spacing configuration of the Advisor HD Grid mapping catheter provides greater VT substrate definition of the BZ particularly at the low-voltage range. This greater resolution within the low-voltage range facilitates CC definition and quantification, essential in guiding ablation strategy [6].

The purpose of this study was to investigate whether VT ablation long-term outcomes were improved with the use of high-density mapping combining complementary mapping strategies into a strict mapping and ablation workflow.

## Methods

Patients who underwent consecutive catheter ablation for VT with structural heart disease from January 2016 to December 2019 at the University Hospital Coventry & Warwickshire (UHCW) were included in this single center study. All adult patients (≥ 18 years) were listed having been discussed at an arrhythmia multidisciplinary team meeting. Clinical indications for VT ablation included symptomatic VT despite optimized medical therapy, three or more episodes of VT within 24 h, at least 3 episodes of VT requiring anti-tachycardia pacing (ATP), or at least one appropriate implantable cardiac defibrillator (ICD) shock. All patients provided written consent prior to the procedure. Approval for the study was provided by our Local Audit and Research Department. The study applied the principles of the Declaration of Helsinki.

### VT mapping protocol

**Fig. 1:**
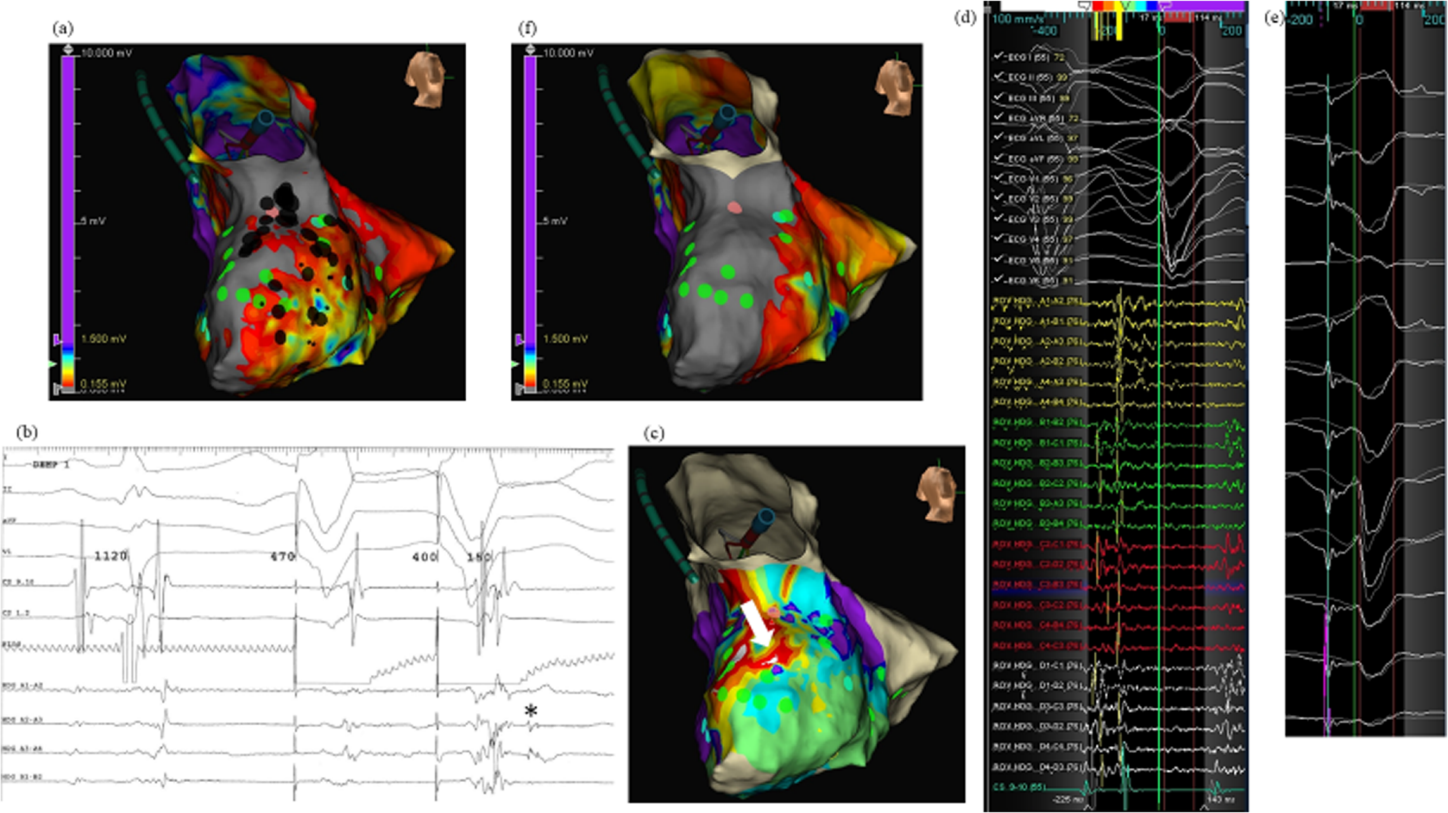
Patient with ischemic cardiomyopathy. VT tachycardia cycle length (TCL) 380 ms mapped using HD Grid. **a** Endocardial LV substrate map demonstrates posterior wall scar with heterogeneous scar extending towards the septum. Voltage scanning (0.155 mV) identifies a CC extending from the septal BZ into the posterior dense scar region. DEEP potentials tagged green. **b** Example DEEP potentials (asterisks) identified with pacing from the right ventricular apex with 2 ventricular sensed extra stimuli at 400 ms. **c** Clinical VT activation map: earliest activation at septal BZ, and with HD Grid positioned at the posteroseptal BZ (sold white arrow); **d** the entire diastolic isthmus activation is localized to this region. HD Grid positioned with D-spine posterior, A-spline septal, and EGMs show diastolic wavefront propagation from posterior dense scar to inferoseptal BZ. **e** Pace mapping at this site matches 12/12, 90% morphology match. Ablation delivered to the posteroseptal BZ targeting the CC and VT isthmus (**a** black tags). **f** Post-ablation substrate re-map confirms CC elimination and homogenization of posteroseptal BZ. Complete procedural success. Pink location tags in **a**, **c**, and **f**: VT “bump” termination with catheter

All cases were first time procedures carried out according to a strict UHCW VT ablation workflow (illustrated in Fig. 1) which systematically applies multiple complimentary mapping strategies described in detail below and published previously [14]. All anti-arrhythmic drugs including amiodarone were discontinued for at least 5 days prior to ablation if the stability of arrhythmia allowed it. The procedure was performed under deep sedation with direct arterial blood pressure and oxygen saturation monitoring with selective procedures performed under general anesthesia (GA) when procedural risk was deemed high as a result of risk markers such as concomitant respiratory disease, obesity, and/or LV ejection fraction (EF) < 25%. Imaging was performed in all patients prior to the procedure to rule out the presence of intra-cardiac thrombus. All patients were administered intravenous unfractionated heparin to maintain an activated clotting time of ≥ 250 s prior to left ventricle (LV) access. Endocardial access to the LV was obtained via transseptal access and retrograde aortic approach in patients as previously described [14]. Epicardial access was obtained in selected cases using the Sosa sub-xiphoid approach with a Tuohy peridural needle (Perican, Melsungen, Germany) prior to systemic anticoagulation [15]. The pericardial puncture was guided by a 90° left lateral fluoroscopic projection. The decision for an epicardial approach was based on underlying heart condition, the VT QRS morphology in a 12-lead ECG, failure of a prior endocardial attempt to abolish the VT, or no dense confluent scar revealed by endocardial voltage mapping. All arrhythmogenic right ventricular cardiomyopathy (ARVC) cases were diagnosed using the International Task Force Criteria [16] and underwent first time epicardial mapping.

Electro-anatomical substrate mapping was performed using the EnSite NAVX/Velocity/Precision (Abbott Medical, Inc., Minneapolis, MN) system or the CARTO (Biosense Webster Inc., Diamond Bar, CA) system. Signals were sampled at 1 kHz and filtered at 0.1 to 50 Hz for surface ECGs and 30 to 250 Hz for intracardiac signals, displayed at an amplification of 0.1 mV/cm. Mapping catheters used were assigned by operator preference to include PbyP RF mapping catheters (Tacticath (Abbott Medical, Inc., Minneapolis, MN), Smart Touch (Biosense Webster, Inc.)), and 3 multipolar catheters: Livewire Duodeca (Abbott Medical, Inc., Minneapolis, MN) which is a 20 electrode, 1 mm 2-2-2 interspacing linear catheter; Pentaray NAV (Biosense Webster, Inc.); and Advisor™ HD Grid mapping catheter (Abbott Medical, Inc., Minneapolis, MN). The Pentaray is a star-shaped catheter with five flexible splines containing 20 electrodes. Two configurations of the catheter are available with varying distances between the electrodes, 6-2-6 and 4-4-4 mm, and in this study, all Pentaray cases were performed with the 4-4-4 mm configuration. The HD Grid has equidistant spacing of 16, 1 mm electrodes in a 4 × 4, 3 mm interspaced arrangement. All HD Grid cases were performed with the Wave configuration [6]. In endocardial cases, the large curve Agilis (St. Jude Medical, St. Paul, MN) steerable sheath was used in combination with the multipolar mapping catheters and the Epi-Agilis (St. Jude Medical, St. Paul, MN) steerable sheath was used in all epicardial cases.

Regular bipolar voltage definitions were used to describe preserved myocardial voltage (> 1.5 mV), BZ voltage (0.5–1.5 mV), and dense scar (< 0.5 mV) [17]. Electrograms (EGMs) were classified according to the standard criteria [18] as follows: (1) normal (≤ 3 sharp intrinsic deflections from baseline, amplitude ≥3 mV, duration < 70 ms, and amplitude: duration > 0.046 mV/ms), (2) fractionated (multiple intrinsic deflections, amplitude ≤ 0.5 mV, duration ≥ 133 ms, and amplitude: duration ≤ 0.005 mV/ms), (3) late (isolated component ≥ 20 ms after the end of surface QRS), and (4) very late potentials (isolated component ≥ 100 ms after the end of surface QRS). CCs were defined as (1) conducting corridors of voltage difference detected by “voltage scanning” at different voltage thresholds within the scar area. The surface bipolar voltage settings were adjusted by gradually reducing the upper threshold cutoff from 1.5 to 0.5 mV in 0.02 mV decrements. Below 0.5 mV, the difference in the upper and lower voltage thresholds was altered keeping a 0.02 mV difference until complete loss of any voltage gradient was achieved [3, 6]; (2) Decrementing evoked potential (DEEP) channel mapping as previously described [9]. To nullify the artifact of epicardial fat and coronary vasculature on epicardial signals, we defined abnormal epicardial electrograms as signals with durations of > 80 ms, demonstrating fractionation with ≥ 2 components, or demonstrating late potentials with an onset well after the QRS [19].

If VT was hemodynamically stable, defined by a systolic blood pressure greater than 90 mmHg, a conventional activation map with a window of interest 90% of the VT cycle length was constructed. When technically feasible, the activation map was turbo-mapped to generate a diastolic activation map to define areas of slow conduction or with the use of ripple mapping. This was followed by entrainment maneuvers [20]. For hemodynamically unstable VT, limited activation maps were attempted by placing the mapping catheter at potential VT isthmus sites prior to VT induction protocols. If a surface 12-lead ECG of the clinical VT was available pre- or peri-procedure, pace mapping was performed with good pacemaps defined as ≥ 11/12 matching leads or > 90% morphology match.

### Ablation parameters

Ablation was performed using either the Smart Touch ablation catheter or the Tacticath ablation catheter. All PbyP mapping cases were performed with either the Smart Touch or Tacticath ablation catheters. The Smart Touch ablation catheter was used in all Pentaray mapping cases. The Tacticath ablation catheter was used in all Duodeca and HD Grid mapping cases. Importantly, ablation parameters were kept consistent between cases with power titrated between 30 and 50 W and an irrigation rate up to 30 mL/min aiming for minimum impedance drops of 15 Ω/lesion. Force time integral ranges were kept between 300 and 400. Ablation targets were limited to CCs and clinical VT critical isthmus sites if entrainment and activation mapping was performed. All epicardial ablation was performed at 30 to 40 W irrigated RF, post-coronary angiography to ensure ablation targets >1 cm from major epicardial coronary arteries and phrenic nerves.

### Post-ablation re-map

A key part of the UHCW VT ablation workflow is the substrate re-map post-ablation using the index mapping catheter to ensure substrate modification. Where viable myocardium was detectable at ablation targets in the re-map, as per “voltage scanning” described above, further ablation was performed to ensure complete substrate elimination. Post-procedure unfractionated heparin was reversed with 50 mg intravenous protamine and for epicardial cases 40 mg triamcinolone/40 mg gentamicin was administered intrapericardial prior to sheath removal.

### Programmed electric stimulation

We used programmed electric stimulation (PES) from the right ventricular apex and outflow tract with 2 different drive cycle lengths (600 and 400 ms) and introduction of up to 3 extrastimuli until a ventricular effective refractory period or a coupling interval of 200 ms was reached, without the use of isoproterenol. Clinical VT was determined from the available 12-lead ECG or VT cycle length in the ICD memory. All other monomorphic VTs including polymorphic VT were deemed non-clinical. Far-field EGM morphology was not routinely taken into consideration to distinguish the VT morphology. Non-clinical monomorphic and hemodynamically stable VTs inducible during the procedure were also targeted for ablation. Complete elimination of any clinical and non-clinical monomorphic VT was defined as complete success. Elimination of the clinical VT only with inducible non-clinical monomorphic VT was defined as partial success. Re-induction of clinical VT despite ablation was defined as procedure failure. Major procedure-related complications were defined as those necessitating additional interventions and leading to prolonged hospitalization.

### Device re-programming and antiarrhythmic drug therapy

To minimize bias, all ICDs were programmed post-procedure according to a standardized protocol on the basis of the best evidence available at the time of study initiation study [21]. In addition, all patients remained on the previously ineffective antiarrhythmic drug therapy after the procedure.

### Clinical follow-up

Long-term follow-up was assessed from the first VT ablation procedure until the latest documented clinical contact which included ICD interrogation data. Patients were followed up at 1, 3, 6, and 12 months for the first year and every 6 months thereafter. ICDs were interrogated at each visit, and arrhythmia logs were retrieved. A detailed history, Holter monitoring, and ECG were performed in symptomatic patients without ICD shocks. VT recurrence was defined as any episode of VT requiring ICD therapy (ATP or shock). Episodes of asymptomatic non-sustained VT (NSVT) recorded from device interrogations were included in the analysis as well as all-cause mortality.

### Statistical analysis

Continuous variables are expressed as mean ± SD. Statistical significance was assessed using the unpaired Student’s *t* test or Mann-Whitney test if necessary. For the comparison of the 4 catheter groups, we used non-parametric Kruskal-Wallis test to compare continuous variables. Categorical variables, expressed as numbers or percentages, were analyzed using the chi-square test, Fisher’s exact test, or McNemar test for paired comparison. Univariate analysis of variables was performed. Cumulative event rates were calculated according to the Kaplan-Meier method. Hazard ratios with corresponding 95% confidence intervals (CIs) are presented. *p* value < 0.05 defined statistical significance. Statistical analysis was performed using MedCalc and Statview 5.0 statistical software.

## Results

On the basis of the above definitions, a total of 73 patients underwent VT ablation with a mean total follow-up of 372 ± 234 days. A total of 33 procedures were performed with the HD Grid mapping catheter, 22 Pentaray, 12 Duodeca, and 6 PbyP mapping cases. The baseline characteristics shown in Table 1 show no significant differences in gender, age, LVEF, or ischemic heart disease etiology between the 4 groups. Importantly, there was a similar proportion of patients suffering with pre-ablation ATP or appropriate shocks with no significant difference in number of baseline clinical VTs. There were no significant differences in co-morbidities or treatment with beta-blockers and/or amiodarone between the 4 groups.

**Table 1:** Baseline clinical and demographic characteristics of the study population comparing HD Grid, Pentaray, Duodeca, and PbyP RF mapping catheter cases

		HD Grid *n* = 33	Pentaray *n* = 22	Duodeca *n* = 12	RF *n* = 6	*p* value
Male, *n* (%)		30 (91)	17 (77)	11 (92)	5 (83)	0.48
Age at ablation, mean ± SD		68.7 ± 11.1	65.2 ± 11.0	72.9 ± 6.9	58.2 ± 16.4	0.05
Etiology	Ischemic	24 (73)	17 (77)	7 (58)	3 (50)	0.46
	DCM	5 (15)	3 (14)	1 (8)	2 (33)	0.57
	HCM	0	2 (9)	3 (25)	0	0.03
	ARVC	4 (12)	0	0	1 (17)	0.18
	Other	0	0	1 (8)	0	0.16
ICD, *n* (%)		32 (97)	21 (95)	12 (100)	4 (67)	0.02
Pre-ablation ATP, *n* (%)		25 (76)	20 (91)	11 (92)	4 (67)	0.28
Pre-ablation shock, *n* (%)		22 (67)	20 (91)	12 (100)	4 (67)	0.07
AF, *n* (%)		11 (33)	8 (36)	8 (67)	1 (17)	0.13
Flutter, *n* (%)		0	0	1 (8)	0	0.16
Hypertension, *n* (%)		16 (48)	14 (64)	4 (33)	1 (17)	0.14
Diabetes Mellitus, *n* (%)		6 (18)	4 (18)	0	0	0.28
Stroke, *n* (%)		1 (3)	0	0	0	0.75
LV dysfunction, *n* (%)		30 (91)	20 (91)	11 (92)	5 (83)	0.94
Ejection Fraction		35.2 ± 11.6	32.3 ± 8.8	31.5 ± 11.5	34 ± 18.2	0.72
Beta-blocker, *n* (%)		33 (100)	21 (95)	11 (92)	6 (100)	0.43
Amiodarone, *n* (%)		25 (76)	19 (86)	8 (67)	5 (83)	0.57
Verapamil, *n* (%)		0	0	0	0	1
Sotalol, *n* (%)		0	0	0	0	1
Flecainide, *n* (%)		0	0	0	0	1
Mexilitine, *n* (%)		0	2 (9)	1 (8)	3 (50)	0.008
Anticoagulation, *n* (%)	Warfarin	5 (15)	11 (50)	5 (42)	1 (33)	0.04
	Apixaban	6 (18)	2 (9)	3 (25)	0	0.41
	Edoxaban	2 (6)	0	8	0	0.56
	Dabigatran	0	1 (4.5)	0	0	0.50
	Rivaroxaban	4 (12)	1 (4.5)	0	0	0.40
Number of clinical VTs, mean ± SD		1.39 ± 0.55	1.50 ± 0.86	1.08 ± 0.3	1.00 ± 0.00	0.14

*DCM* dilated cardiomyopathy, *HCM* hypertrophic cardiomyopathy, *AVC* arrhythmogenic cardiomyopathy, *ICD* implantable cardiac defibrillator, *ATP* anti-tachycardia pacing, *AF* atrial fibrillation

### Procedural data

Procedural data recorded for each mapping catheter has been summarized in Table 2. The majority of cases in all 4 groups were performed under deep sedation with endocardial LV mapping. There was no significant difference in the proportion of patients undergoing endocardial RV mapping. All epicardial mapping cases were performed with multipolar mapping catheters.

**Table 2:** Electrophysiological and procedural characteristics of the study population

		HD Grid *n* = 33	Pentaray *n* = 22	Duodeca *n* = 12	RF *n* = 6	*p* value
GA, *n* (%)		6 (18)	6 (27)	2 (17)	1 (17)	0.83
RV mapping, *n* (%)		7 (21)	3 (14)	3 (25)	1 (17)	0.71
LV mapping, *n* (%)		31 (94)	22 (100)	12 (100)	5 (83)	0.24
Endocardial mapping, *n* (%)		33 (100)	21 (95)	12 (100)	6 (100)	0.50
Epicardial mapping, *n* (%)		7 (21)	1 (4)	2 (16)	0	0.24
Epicardial access, *n* (%)		7 (21)	1 (4)	1 (8)	0	0.20
Pre-ablation PES, *n* (%)		9 (27)	5 (23)	6 (50)	5 (83)	0.02
VT hemodynamically stable, *n* (%)		18 (55)	12 (55)	8 (67)	3 (50)	0.88
VT CL ms, mean ± SD		364 ± 68	382 ± 86	371 ± 54	358 ± 65	0.91
Substrate map, *n* (%)		33 (100)	22 (100)	11 (92)	5 (83)	0.06
Activation map, *n* (%)		27 (82)	14 (64)	11 (92)	2 (33)	0.025
Pace map, *n* (%)		20 (61)	11 (50)	11 (92)	4 (67)	0.11
Pace map >11/12, *n* (%)		19 (58)	10 (45)	10 (83)	1 (17)	0.039
Entrainment map, *n* (%)		7 (21)	5 (23)	4 (33)	0	0.45
RF catheter	Smart Touch, *n* (%)	0	22 (100)	0	4 (66)	< 0.001
	Tacticath, *n* (%)	33 (100)	0	12 (100)	2 (33)	< 0.001
VTs induced/case, mean ± SD		2.1 ± 0.4	1.9 ± 1.1	1.8 ± 0.5	1.3 ± 0.8	0.11
VTs mapped, mean ± SD		1.5 ± 0.7	1.7 ± 1.46	1.25 ± 0.45	0.5 ± 0.5	0.06
VTs ablated, mean ± SD		1.4 ± 0.7	1.6 ± 1.46	1.25 ± 0.45	0.5 ± 0.5	0.09
Initial substrate map time (min), mean ± SD		37 ± 10.9	63 ± 20.2	55 ± 16	96 ± 23.6	< 0.001
Number of maps/case, mean ± SD		2.3 ± 0.68	1.3 ± 0.46	1.5 ± 0.52	1 ± 0	< 0.001
Mapping points/map, mean ± SD		2687 ± 2192	1426 ± 1192	1256 ± 892	207 ± 202	< 0.001
Post-procedure clinical VT inducible, *n* (%)		1 (3)	4 (18)	2 (17)	3 (50)	0.016
Post-procedure non-clinical VT inducible, *n* (%)		7 (21)	9 (41)	1 (8)	4 (67)	0.027
Elimination of all clinical & non-clinical VTs *n*,(%)		26 (79)	12 (55)	10 (83)	2 (33)	0.04
Complications, *n* (%)		2 (6)	2 (9)	1 (8)	1 (17)	0.68
RF total (min), mean ± SD		42 ± 19	62 ± 18	34 ± 18	29 ± 10	0.001
Fluoroscopy time (min), mean ± SD		34.5 ± 14	28.5 ± 8.3	31.9 ± 6.3	30.3 ± 11.3	0.31
Procedure time (min), mean ± SD		350 ± 105	261 ± 59	257 ± 74	239 ± 72	< 0.001
Post procedure echo, *n* (%)		33 (100)	21 (95)	12 (100)	6 (100)	0.50

*GA* general anesthesia, *PES* pulsed electrical stimulation, *CL* cycle length

Substrate mapping was performed in 97% (71/73) of cases with no significant difference between the 4 mapping groups. There was no significant difference in the frequency of mappable hemodynamically stable VTs between the 4 groups; however, activation maps used to guide ablation were successfully generated in 82% of HD Grid cases, 64% of Pentaray cases, 92% Duodeca cases, and only 33% of RF mapping cases (*p* = 0.025). A 2 × 2 analysis between groups showed no difference between the HD Grid, Pentaray, or Duodeca catheters; however, both HD Grid (*p* = 0.04) and Duodeca (*p* = 0.04) were superior to PbyP RF catheter activation mapping. A similar trend was observed with successful entrainment mapping. There was no significant difference in the frequency of pace mapping performed between the 4 groups; however, successful pacemaps used to guide ablation were more likely to be obtained with the multipolar mapping catheters (HD Grid 58%; Pentaray 45%, Duodeca 83%) than the PbyP RF mapping group (17%; *p* = 0.039). Accordingly, a greater number of VTs were mapped with multipolar catheters and thereafter ablated. There was a trend towards a higher number of VTs mapped and subsequently ablated in the HD Grid and Pentaray groups.

The mean initial substrate mapping time of the chamber of interest was shortest for the HD Grid compared to the Pentaray, Duodeca, and PbyP RF groups (37 ± 10.9 min, 63 ± 20.2 min, 55 ± 16 min, 96 ± 23.6 min; *p* < 0.001), with no significant difference between the Pentaray and Duodeca groups. Importantly, there was no difference in frequency of LV or RV endocardial mapping between groups. The mean number of maps constructed per case excluding the Precision One Map and Turbomap functions was greatest in the HD Grid group (2.3 ± 0.68; *p* < 0.001), with no significant difference between the Pentaray (1.3 ± 0.46) and Duodeca (1.5 ± 0.52) groups and the number of points used per map were greater with HD Grid than any other mapping catheter. There was a significantly greater duration of RF delivery in the multipolar mapping groups than the PbyP RF group and fluoroscopy times were similar between all 4 groups.

In terms of post-procedural endpoint testing, a significantly greater number of clinical VTs were still inducible in the PbyP RF mapping group (50%, *p* = 0.016) compared to the multipolar mapping catheters (HD Grid 3%, Pentaray 18%, Duodeca 17%). Complete elimination of clinical and non-clinical VTs was achieved in 79% of HD Grid, 55% of Pentaray, 83% Duodeca, and 33% of PbyP RF mapping cases (*p* = 0.04) with no statistical difference with comparative analysis between the HD Grid, Pentaray, or Duodeca cases.

There was no significant difference in complication rates between the 4 groups. In total, 2 patients suffered pericardial effusions, one of which required a pericardial drain. There were 2 patients that suffered femoral vascular complications, one with a groin hematoma, and one patient with a pseudoaneurysm. One patient suffered pulmonary edema and one patient developed left bundle branch block necessitating device upgrade to biventricular pacing.

### Long-term outcome

**Fig. 2:**
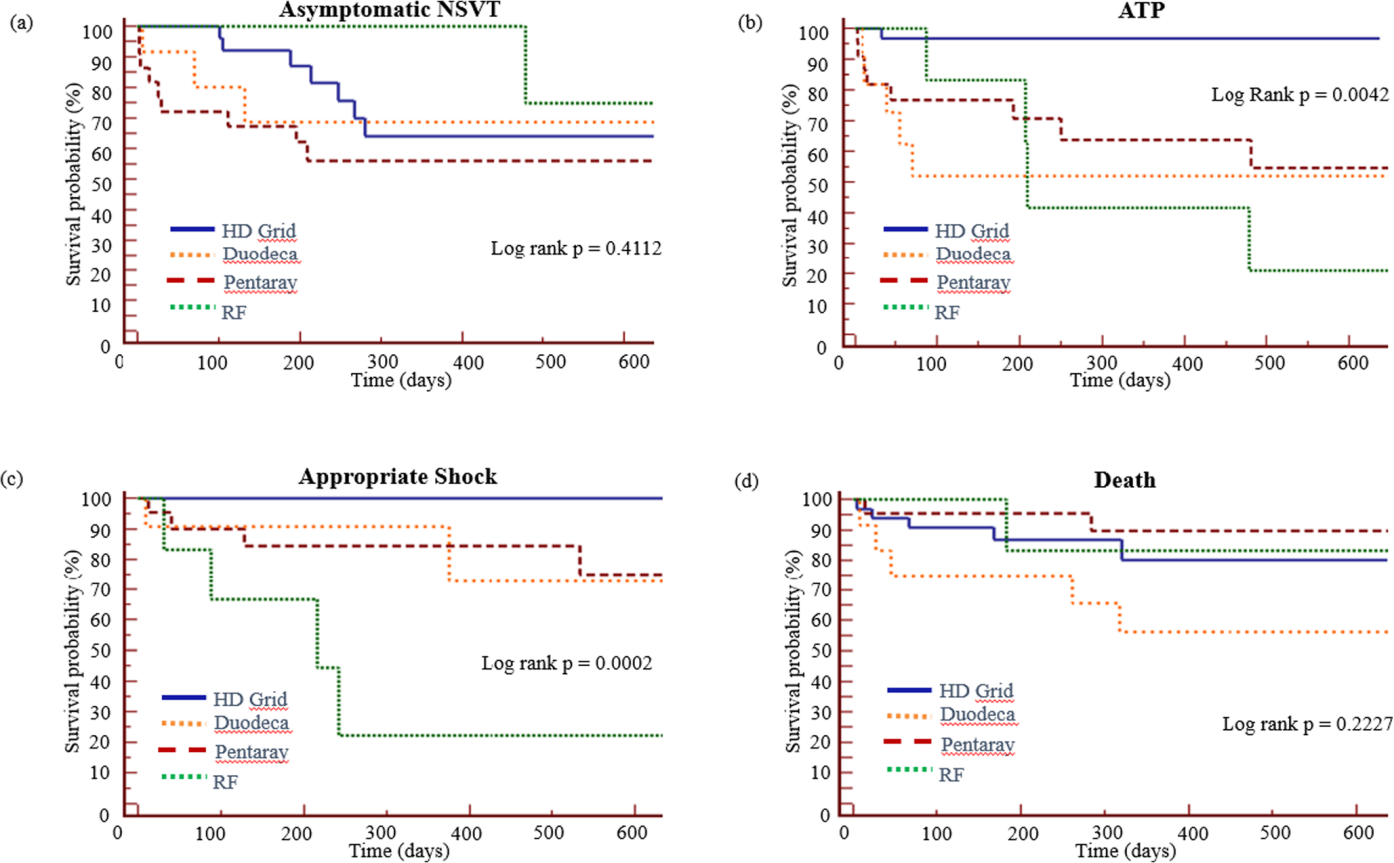
Kaplan-Meir curves of all 4 mapping catheters showing event free survival from **a** asymptomatic NSVT, **b** anti-tachycardia pacing (ATP), **c** appropriate shock, and **d** death

Figure 2 shows the event-free survival from (a) asymptomatic NSVT, (b) ATP, (c) appropriate shocks, and (d) death in all 4 mapping catheter groups. There was no statistical difference in asymptomatic NSVT. In terms of ATP (2b) and appropriate ICD shock (2c), there was a step-wise improvement in event-free survival from PbyP RF catheter mapping (33% ATP-free, 33% shock-free) to multipolar mapping with the Pentaray (64% ATP-free, 82% shock-free) and Duodeca (58% ATP-free, 83% shock-free) with the lowest event rates in the HD Grid group (97% ATP-free, 100% shock-free). No difference in mortality was observed.

There was a 96% relative reduction in device therapy for ATP in the HD Grid group as 25/33 (76%) patients received ATP pre-ablation compared to 1/33 (3%) at final follow-up. In the Pentaray, Duodeca, and PbyP RF groups, 20/22 (91%), 11/12 (92%), and 4/6 (67%) patients received ATP pre-ablation, compared to 8/22 (36%), 5/12 (42%), and 4/6 (67%) at final follow-up; relative reductions of 60%, 55% and 0% respectively (*p* = 0.004). An analysis of appropriate shocks showed a 100% relative reduction in device therapy for appropriate shock in the HD Grid group as 22/33 (67%) patients received shock therapy pre-ablation compared to 0/33 at final follow-up. In the Pentaray, Duodeca, and PbyP RF groups, 20/22 (91%), 12/12 (100%), and 4/6 (67%) patients received shock therapy pre-ablation compared to 4/22 (18%), 2/12 (17%) and 4/6 (67%) at final follow-up; relative reductions of 80%, 83%, and 0% respectively (*p* = 0.008). As shown in Fig. 2b and c, time to first ATP therapy in the 4 groups were 33, 2, 9, and 87 days, respectively. There were no appropriate shocks in the HD Grid group and time to first shock therapy in the Pentaray, Duodeca, and RF groups were 12, 9, and 31 days respectively.

## Discussion

### Main findings

This study shows that high density grid mapping combining complementary mapping strategies for structural heart VT ablation provides a significant advantage in long-term outcomes. The rationale for this analysis, of comparing the unique ability of the HD Grid mapping catheter to detect orthogonal low amplitude voltages, was based on our previous reports of an increase in CCs identifiable when compared to a high density linear multipolar catheter configuration [6]. The main findings of this study are (1) multipolar mapping catheters facilitate mapping an increased number of hemodynamically stable and unstable VTs compared to PbyP RF catheter mapping, (2) the greater number of VTs mapped with the HD Grid catheter corresponds to an increased number of clinical and non-clinical VTs ablated, (3) the high-density maps created with the HD Grid catheter correlates not only with improved procedural endpoints but with long-term clinical outcomes, and (4) HD Grid mapping provides an advantage in long-term survival free of ICD therapies.

### Diversity of multipolar mapping

An important determinant of successful VT ablation is an informative substrate map and we have shown the capability of multipolar and PbyP RF catheters in generating an initial substrate map with significant differences in map construct time. The time taken to generate an initial substrate map with the HD Grid catheter was 37 ± 10.9 mins compared to 96 ± 23.6 with PbyP RF catheters (*p* < 0.001). Our experience with the Pentaray suggests that the irritability of the 5 open-ended splines can result in longer map times due to the requirement of a 95% sinus rhythm or paced rhythm morphology template (identical set-up with HD Grid and Duodeca cases). A previous study in structural heart VT patients demonstrated similar map times of 55.6 ± 34.4 min with the Pentaray [22]. Despite a shorter initial substrate mapping time, the procedural time was significantly longer in the HD Grid group compared to other mapping catheters due to a combination of factors. There was a trend towards a greater number of clinical VTs and VTs inducible in the multipolar mapping catheter groups, an increase in RF time in the HD Grid group compared to the PbyP and Duodeca groups and almost twice as many maps per case constructed with the HD Grid group compared to all other mapping catheters. In addition, the initial cases performed with HD Grid involved a learning curve which resulted in the prolonged procedure times substantiated by the larger standard deviation 350 ± 105 mins.

We have shown a clear distinction in the ability of multipolar mapping catheters to generate “usable” activation maps. This requires the catheter to be manipulated rapidly during VT with the acquisition of point density sufficient enough to define the VT isthmus, which can be compromised with limited point acquisition with PbyP RF catheter mapping. Furthermore, the ability of pacemapping to be performed from multiple sites without having to manipulate the multipolar mapping catheter may explain why these catheters were more effective in generating good pacemaps when compared to PbyP RF catheters. It has previously been shown that the use of multipolar catheters to focus high-density mapping is an independent predictor of VT-free survival [23, 24]. Importantly, the multipolar mapping catheter used in these previous studies was the Pentaray and our study highlights the additional benefits of mapping with the HD Grid.

A key part of the UHCW VT ablation workflow is the re-map performed post-ablation to ensure substrate elimination. The increased number of maps constructed per case with the HD Grid catheter emphasizes its advantage in this regard in terms of speed and detail of map construction to guide further ablation if necessary. It is plausible that the process of re-mapping to further target abnormal electrograms may have in part contributed to the improved outcomes of the HD Grid catheter.

### Impact on procedural endpoints and long-term clinical outcomes

Successful elimination of all clinical and non-clinical VTs was more likely to be achieved with ablation guided by multipolar mapping catheters. The improvement in ICD outcomes in the HD Grid group compared with the Pentaray and DuoDeca groups appears to have been driven by the near absence of clinical VTs inducible at endpoint testing within the HD Grid group (3%). It is plausible that the lower numbers of non-clinical VTs inducible at endpoint testing in the Duodeca group (8%) as compared to the HD Grid (21%), Pentaray (41%), and PbyP (66%) groups was a chance effect and this may have resulted in the higher than expected rate of complete elimination of all clinical and non-clinical VTs observed in the Duodeca group (83%).

There was a significant reduction in the duration of RF delivered in PbyP RF cases compared to multipolar mapping cases and this may have been as a result of fewer ablation targets identifiable in the RF group, despite no difference in LV EF and VT substrate. This resulted in shorter procedure times with the PbyP RF mapping group. It is plausible that the ablation delivery to HD Grid mapped substrate is targeted more effectively towards the arrhythmogenic substrate.

HD Grid mapping resulted in a significant reduction in ATP and/or appropriate shocks compared to other mapping catheters. We observed no difference in device detected rates of asymptomatic NSVT or all cause death. Comparison of long-term outcomes based on mapping catheter type shows a step-wise improvement in freedom from recurrent ATP or shock, such that multipolar mapping with the Pentaray or Duodeca catheter was superior to PbyP RF catheter mapping, whereas mapping with the HD Grid portended the best long-term results. The explanation for this step-wise improvement in outcome is likely to be multifactorial and due to the unique electrode grid design that facilitates rapid high density mapping and mitigates the effects of wavefront propagation on local electrogram amplitude and characteristics [13, 25].

Indeed, in 13 large VT ablation [8, 26–37] studies the type of mapping catheter is mentioned in the methodologies in 8 studies [8, 28, 30, 31, 33–35, 37] and only 2 of these studies [8, 30] mentioned the use of multipolar mapping catheters. Various mapping strategies are described with studies using exclusive or combinations of substrate, entrainment, and pacemapping as well as targeting LAVA and DEEP potentials, resulting in a range of acute procedural success rates from 41 to 94%. More recently, the use of high-density mapping catheters in defining VT isthmus sites using a novel re-entry vulnerability index (RVI) has been described. Despite this index correlating with pacemap and entrainment map defined VT isthmus sites in 72% of VTs, 16 month follow-up resulted in a 50% recurrence rate [38]. The advantages of HD Grid mapping versus PbyP RF mapping in focal arrythmias with normal structural hearts has been recently highlighted, with significantly earlier electrograms at the site of successful ablation and a significant reduction in map and procedure times [39]. However, neither of these positive outcomes impacted on acute procedural or long-term success. A smaller study of 22 patients evaluating the HD Grid catheter in ventricular arrythmias including 15 patients with structural heart disease VT reported acute procedural success rates of 86% [40]. Patients underwent short-term follow-up at a median of 145 days with a total cohort ventricular arrhythmia recurrence of 27%. The authors did not report short-term outcomes specifically for the structural heart disease patients. A strength of our study is the systematic application of the UHCW VT ablation workflow which applies complementary multiple mapping strategies using high density mapping catheters which resulted in 29/73 (40%) patients suffering recurrent ATP, shock or death in the total cohort, compared to only 6/33 (18%) in the HD Grid group, with long-term follow-up at a mean of 12 ± 8 months (372 ± 234 days). A total of 14 patients died during the follow-up period (HD Grid 5; Pentaray 3; Duodeca 5; RF 1). One death was arrhythmic, 24 days post-ablation in a patient with hypertrophic cardiomyopathy (EF 22%) who underwent combined endocardial and epicardial ablation with complete elimination of all on-table VTs. The remainder included 6 non-cardiac and 7 deaths due to progressive heart failure.

The long-term outcome for patients undergoing VT ablation targeting late potentials varies depending on the scar substrate. Late potentials are more commonly observed in ischemic cardiomyopathy (ICM) than in non-ischemic cardiomyopathy (NICM), and it has been shown that approaches incorporating late potential ablation and pace mapping have limited success in patients with NICM compared with ICM [41, 42] . This indeed may incur potential bias and limitations of HD Grid substrate mapping in NICM. The number of patients with NICM within our study was small and we did not observe a difference in outcome within HD Grid mapped NICM cases compared to ICM outcomes. A potential explanation is that the UHCW mapping strategy is based on DEEP potential mapping and VT site of origin (critical isthmus) mapping which differed from the substrate mapping approaches described by Nakahara et al. Furthermore, the mapping catheters used by Nakahara et al. were limited to PbyP Navi-Star catheters (3.5 or 4 mm tip electrode, Biosense-Webster) and multipolar mapping with the Duodeca catheter. The HD Grid provides high density sampling, improved signal resolution afforded by small closely spaced bipolar recordings that minimizes the need for catheter positioning, with the unique electrode grid design that mitigates the effects of wavefront propagation on local electrogram amplitude and characteristics. These catheter properties would additionally contribute to the improved outcomes in NICM patients. Our overall cohort survival rates free of ATP or shock are similar to previously reported studies; however, we have shown a significant improvement with HD Grid mapping strategies.

### Limitations

The main limitation of our paper is that the study population was not randomized. We did not perform a qualitative assessment of the substrate and activation maps obtained with the different mapping catheters. Such comparison would have required each patient to undergo mapping with all 4 mapping catheters and a detailed analysis of maps generated with identical substrate. This comparison, however ideal, would have unnecessarily increased procedure time and was economically unviable.

The field of VT mapping and ablation has grown exponentially with novel methods of identifying the VT site of origin with newer technologies. Methods such as RVI, MRI-assisted channel identification, and non-contact mapping technologies were not assessed in this study.

Finally, an assessment of lesion durability such as comparisons of impedance change, force-time integral, or ablation lesion indices were not performed. This important aspect of VT mapping and ablation forms the basis of further investigation.

## Conclusions

A wide variety of mapping catheters can be used with an armamentarium of strategies to define VT ablation targets. We have shown a step-wise improvement in survival free from ICD therapies as the density of mapping capability increases. By using a high-density mapping catheter and combining complementary mapping strategies in a strict workflow, long-term clinical outcomes are improved.

## References

[1] Di Biase L, Burkhardt JD, Lakkireddy D, Carbucicchio C, Mohanty S, Mohanty P (2015). Ablation of stable VTs versus substrate ablation in ischemic cardiomyopathy the VISTA randomized multicenter trial. J Am Coll Cardiol.

[2] Proietti R, Essebag V, Beardsall J, Hache P, Pantano A, Wulffhart Z (2014). Substrate-guided ablation of haemodynamically tolerated and untolerated ventricular tachycardia in patients with structural heart disease: effect of cardiomyopathy type and acute success on long-term outcome. Europace.

[3] Jamil-Copley S, Vergara P, Carbucicchio C, Linton N, Koa-Wing M, Luther V (2014). Application of ripple mapping to visualize slow conduction channels within the infarct-related left ventricular scar. Circ Arrhythm Electrophysiol.

[4] Proietti R, Roux J-F, Essebag V (2016). Recent advances in ablation of ventricular tachycardia associated with structural heart disease: overcoming the challenges of functional and fixed barriers. Curr Opin Cardiol.

[5] Proietti R, Roux J-F, Verma A, Alturki A, Bernier ML, Essebag V (2016). A historical perspective on the role of functional lines of block in the re-entrant circuit of ventricular tachycardia. PACE-Pacing Clin Electrophysiol.

[6] Proietti R, Adlan AM, Dowd R, Assadullah S, Aldhoon B, Panikker S (2019). Enhanced ventricular tachycardia substrate resolution with a novel omnipolar high-density mapping catheter: the omnimapping study. J Interv Card Electrophysiol.

[7] Bricenõ DF, Romero J, Villablanca PA, Londonõ A, Diaz JC, Maraj I (2018). Long-term outcomes of different ablation strategies for ventricular tachycardia in patients with structural heart disease: systematic review and meta-analysis. Europace..

[8] Jaïs P, Maury P, Khairy P, Sacher F, Nault I, Komatsu Y (2012). Elimination of local abnormal ventricular activities: a new end point for substrate modification in patients with scar-related ventricular tachycardia. Circulation..

[9] Porta-Sánchez A, Jackson N, Lukac P, Kristiansen SB, Nielsen JM, Gizurarson S (2018). Multicenter study of ischemic ventricular tachycardia ablation with decrement-evoked potential (DEEP) mapping with extra stimulus. JACC Clin Electrophysiol.

[10] Verma A, Marrouche NF, Schweikert RA, Saliba W, Wazni O, Cummings J (2005). Relationship between successful ablation sites and the scar border zone defined by substrate mapping for ventricular tachycardia post-myocardial infarction. J Cardiovasc Electrophysiol.

[11] Proietti R, Jacqueline Joza VE (2016). Therapy for ventricular tachycardia in structural heart disease : a multifaceted challenge. J Physiol.

[12] Josephson ME, Anter E (2015). Substrate mapping for ventricular tachycardia assumptions and misconceptions. JACC Clin Electrophysiol.

[13] Tung R, Josephson ME, Bradfield JS, Shivkumar K (2016). Directional influences of ventricular activation on myocardial scar characterization: voltage mapping with multiple wavefronts during ventricular tachycardia ablation. Circ Arrhythm Electrophysiol.

[14] Adlan AM, Arujuna A, Dowd R, Hayat S, Panikker S, Foster W (2019). Long-term follow-up of normal and structural heart ventricular tachycardia catheter ablation : real-world experience from a UK tertiary centre. Open Hear.

[15] Sosa E, Scanavacca M, d’Avila A, Pilleggi E (1996). A new technique to perform epicardial mapping in the electrophysiology laboratory. J Cardiovasc Electrophysiol.

[16] Marcus FI, WJ MK, Sherrill D, Basso C, Bauce B, Bluemke DA (2010). Diagnosis of arrhythmogenic right ventricular cardiomyopathy/dysplasia: proposed modification of the task force criteria. Circulation..

[17] Marchlinski FE, Callans DJ, Gottlieb CD, Zado E (2000). Linear ablation lesions for control of unmappable ventricular tachycardia in patients with ischemic and nonischemic cardiomyopathy. Circulation..

[18] Bogun F, Good E, Reich S, Elmouchi D, Igic P, Lemola K (2006). Isolated potentials during sinus rhythm and pace-mapping within scars as guides for ablation of post-infarction ventricular tachycardia. J Am Coll Cardiol.

[19] Cano O, Hutchinson M, Lin D, Garcia F, Zado E, Bala R (2009). Electroanatomic substrate and ablation outcome for suspected epicardial ventricular tachycardia in left ventricular nonischemic cardiomyopathy. J Am Coll Cardiol.

[20] Stevenson WG, Friedman PL, Sager PT, Saxon LA, Kocovic D, Harada T (1997). Exploring postinfarction reentrant ventricular tachycardia with entrainment mapping. J Am Coll Cardiol.

[21] Wilkoff BL, Williamson BD, Stern RS, Moore SL, Lu F, Lee SW (2008). PREPARE Study Investigators: strategic programming of detection and therapy parameters in implantable cardioverter-defibrillators reduces shocks in primary prevention patients: results from the PREPARE (Primary Prevention Parameters Evaluation) study. J Am Coll Cardiol.

[22] Maagh P, Christoph A, Dopp H, Mueller MS, Plehn G, Meissner A (2017). High-density mapping in ventricular tachycardia ablation: a PentaRay® study. Cardiol Res.

[23] Wolf M, Sacher F, Cochet H, Kitamura T, Takigawa M, Yamashita S (2018). Long-term outcome of substrate modification in ablation of post–myocardial infarction ventricular tachycardia. Circ Arrhythm Electrophysiol.

[24] Yamashita S, Cochet H, Sacher F, Mahida S, Berte B, Hooks D (2016). Impact of new technologies and approaches for post–myocardial infarction ventricular tachycardia ablation during long-term follow-up. Circ Arrhythm Electrophysiol.

[25] Gaeta S, Bahnson TD, Henriquez C (2020). Mechanism and magnitude of bipolar electrogram directional sensitivity: characterizing underlying determinants of bipolar amplitude. Heart Rhythm.

[26] Berruezo A, Fernández-Armenta J, Andreu D, Penela D, Herczku C, Evertz R (2015). Scar dechanneling: a new method for scar-related left ventricular tachycardia substrate ablation. Circ Arrhythm Electrophysiol.

[27] Calkins H, Epstein A, Packer D, Arria AM, Hummel J, Gilligan DM (2000). Cooled RF Multi Center Investigators Group. Catheter ablation of ventricular tachycardia in patients with structural heart disease using cooled radiofrequency energy: results of a prospective multicenter study. J Am Coll Cardiol.

[28] Carbucicchio C, Santamaria M, Trevisi N, Maccabelli G, Giraldi F, Fassini G (2008). Catheter ablation for the treatment of electrical storm in patients with implantable cardioverter-defibrillators : short-and long-term outcomes in a prospective single-center study. Circulation..

[29] Dinov B, Fiedler L, Schönbauer R, Bollmann A, Rolf S, Piorkowski C (2014). Outcomes in catheter ablation of ventricular tachycardia in dilated nonischemic cardiomyopathy compared with ischemic cardiomyopathy: results from the Prospective Heart Centre of Leipzig VT (HELP-VT) Study. Circulation..

[30] Kuck K-H, Schaumann A, Eckardt L, Willems S, Ventura R, Delacrétaz E (2010). Catheter ablation of stable ventricular tachycardia before defibrillator implantation in patients with coronary heart disease (VTACH): a multicentre randomised controlled trial. Lancet..

[31] Kuck K-H, Tilz RR, Deneke T, Hoffmann BA, Ventura R, Hansen PS (2017). Impact of substrate modification by catheter ablation on implantable cardioverter–defibrillator interventions in patients with unstable ventricular arrhythmias and coronary artery disease: results from the multicenter randomized controlled SMS (Substrate). Circ Arrhythm Electrophysiol.

[32] Niwano S, Fukaya H, Yuge M, Imaki R, Hirasawa S, Sasaki T (2008). Role of electrophysiologic study (EPS)-guided preventive therapy for the management of ventricular tachyarrhythmias in patients with heart failure. Circ J.

[33] Reddy VY, Reynolds MR, Neuzil P, Richardson AW, Taborsky M, Jongnarangsin K (2007). Prophylactic catheter ablation for the prevention of defibrillator therapy. N Engl J Med.

[34] Sapp JL, Wells GA, Parkash R, Stevenson WG, Blier L, Sarrazin J-F (2016). Ventricular tachycardia ablation versus escalation of antiarrhythmic drugs. N Engl J Med.

[35] Stevenson WG, Wilber DJ, Natale A, Jackman WM, Marchlinski FE, Talbert T (2008). Multicenter Thermocool VT Ablation Trial Investigators: Irrigated radiofrequency catheter ablation guided by electroanatomic mapping for recurrent ventricular tachycardia after myocardial infarction: The multicenter thermocool ventricular tachycardia abla. Circulation..

[36] Tanner H, Hindricks G, Volkmer M, Furniss S, Kühlkamp V, Lacroix D (2010). Catheter ablation of recurrent scar-related ventricular tachycardia using electroanatomical mapping and irrigated ablation technology: results of the prospective multicenter Euro-VT-study. J Cardiovasc Electrophysiol.

[37] Willems S, Tilz RR, Steven D, Kääb S, Wegscheider K, Gellér L (2020). Preventive or deferred ablation of ventricular tachycardia in patients with ischemic cardiomyopathy and implantable defibrillator (BERLIN VT) a multicenter randomized trial. Circulation..

[38] Orini M, Graham AJ, Srinivasan NT, Campos FO, Hanson BM, Chow A (2020). Evaluation of the reentry vulnerability index to predict ventricular tachycardia circuits using high-density contact mapping. Heart Rhythm.

[39] Chieng D, Lahiri A, Sugumar H, Al-Kaisey A, Parameswaran R, Anderson RD, et al. Multipolar mapping with the high-density grid catheter compared with conventional point-by-point mapping to guide catheter ablation for focal arrhythmias. J Cardiovasc Electrophysiol 2020 10.1111/jce.14636 32583514

[40] Campbell T, Trivic I, Bennett RG, Anderson RD, Turnbull S, Pham T (2020). Catheter ablation of ventricular arrhythmia guided by a high-density grid catheter. J Cardiovasc Electrophysiol.

[41] Nakahara S, Tung R, Ramirez RJ, Michowitz Y, Vaseghi M, Buch E (2010). Characterization of the arrhythmogenic substrate in ischemic and nonischemic cardiomyopathy: implications for catheter ablation of hemodynamically unstable ventricular tachycardia. J Am Coll Cardiol.

[42] Piers SRD, Leong DP, van Taxis CFB v H, Tayyebi M, Trines SA, Pijnappels DA (2013). Outcome of ventricular tachycardia ablation in patients with nonischemic cardiomyopathy: the impact of noninducibility. Circ Arrhythm Electrophysiol.

